# The Use of Electrospun Organic and Carbon Nanofibers in Bone Regeneration

**DOI:** 10.3390/nano10030562

**Published:** 2020-03-20

**Authors:** Kaoru Aoki, Hisao Haniu, Yoong Ahm Kim, Naoto Saito

**Affiliations:** 1 Physical Therapy Division, School of Health Sciences, Shinshu University, 3-1-1 Asahi, Matsumoto, Nagano 390-8621, Japan; 2 Institute for Biomedical Sciences, Interdisciplinary Cluster for Cutting Edge Research, Shinshu University, 3-1-1 Asahi, Matsumoto, Nagano 390-8621, Japan; hhaniu@shinshu-u.ac.jp (H.H.); saitoko@shinshu-u.ac.jp (N.S.); 3 Department of Biomedical Engineering, Graduate School of Medicine, Science and Technology, Shinshu University, 3-1-1 Asahi, Matsumoto, Nagano 390-8621, Japan; 4 Department of Polymer Engineering, Alan G. MacDiarmid Energy Research Institute, Chonnam National University, 77 Yongbong-ro, Buk-gu, Gwangju 61186, Korea; yak@chonnam.ac.kr

**Keywords:** bone defects, scaffolds, electrospinning, bone regeneration, electrospun nanofibers

## Abstract

There has been an increasing amount of research on regenerative medicine for the treatment of bone defects. Scaffolds are needed for the formation of new bone, and various scaffolding materials have been evaluated for bone regeneration. Materials with pores that allow cells to differentiate into osteocytes are preferred in scaffolds for bone regeneration, and porous materials and fibers are well suited for this application. Electrospinning is an effective method for producing a nanosized fiber by applying a high voltage to the needle tip containing a polymer solution. The use of electrospun nanofibers is being studied in the medical field, and its use as a scaffold for bone regeneration therapy has become a topic of growing interest. In this review, we will introduce the potential use of electrospun nanofiber as a scaffold for bone regenerative medicine with a focus on carbon nanofibers produced by the electrospinning method.

## 1. Introduction

Bone tissue repair is essential for the treatment of bone defects resulting from severe fractures, the resection of bone and soft tissue tumor, and spinal fusion surgery [1,2,3]. Various methods are used to compensate for bone defects, including autogenous bone grafting [4,5], allogeneic bone grafting [6,7,8], artificial bone grafting [9,10,11,12,13], bone transport [14,15], and the induced membrane technique [16]. Because normal bone is harvested from osseous donor sites such as the fibula and iliac crest in autogenous bone grafting, operative procedures must be performed on unaffected parts of the body and may induce pain and functional disorders; furthermore, there is a limit to the amount of graft that can be harvested in a patient [17,18,19,20]. On the other hand, an adequate amount of allogeneic bone can be obtained for grafting, provided that the country or region maintains a bone bank [21,22,23,24,25]. However, because allogeneic bone grafts are harvested from donors other than the patient and are thermally treated to suppress an immune response, the use of these “necrotic bone tissues” may cause graft nonunion or resorption that could lead to re-fracturing and revision surgery [26,27,28].

Biphasic calcium phosphates such as β-tricalcium phosphate (βTCP) and hydroxyapatite (HA) are clinically available as synthetic bone substitutes [10,11,12,13]. These substitutes do not function as bone themselves; rather, bone cells first infiltrate the synthetic substitute that eventually gets resorbed by osteoclasts, and osteoblasts then form new bone matrices in place of the synthetic substitute to function as bone tissues [29,30]. Therefore, there is a limitation to the size of the bone defects that the synthetic bone substitute can compensate for.

In bone transport techniques, pins are inserted into the bone both proximally and distally to the bone defect and secured to an external fixator. A corticotomy is performed away from the bone defect, and the defect is filled with newly formed bone tissues by transporting the pins for gradual distraction. Although good autogenous bone formation can be expected, only 0.25–0.5 mm of newly formed bone can be regenerated per day, and further time is required to obtain adequate osseous stability to remove the external fixator [14,15]. In an induced membrane technique, bone cement is implanted into the bone defect and its surroundings to induce the formation of membranes. The bone cement is removed after 6–8 weeks and autologous bone grafting is performed within the induced membrane. Although the technique is used for large bone defects, the amount of regeneration is still limited to approximately 10 cm of defect [31].

For larger defects with bone tissues that are difficult to repair, patients must undergo arthroplasty with cobalt chrome or titanium alloy prostheses. However, unlike the arthroplasties for joint deformity that are performed in the elderly population, many patients with bone tumors or trauma are younger. Young patients undergoing an artificial replacement of the bone or joint are at a greater risk of implant failure or aseptic loosening that may require revision surgery [32]. Revision arthroplasty is more invasive than primary surgery, often involving longer procedures, greater risk of infection, and increased blood loss. Moreover, when bone defects become larger or adhesion to the surrounding tissues become stronger due to revision surgery, the activities of daily living (ADL) can decrease as a result of a decreased range of motion and shorter distances for ambulation. If a large bone defect can be repaired by bone regeneration therapy, the potential risks from the use of megaprostheses can be avoided.

There has been much research in regenerative medicine due to the discovery and development of pluripotent stem cells such as induced pluripotent stem cells (iPS) [33,34]. Similar to the regenerative medicine of other organs and tissues, important components in bone regenerative medicine consist of (1) cells, (2) scaffolds, and (3) signaling molecules. In addition to the cellular differentiation into the tissues and signaling molecules that stimulate its development, scaffolds that provide cellular adhesion/proliferation and maintain/sustain signaling molecules are indispensable for regenerative medicine [35]. There are currently no established methods of bone regeneration that can repair large defects in order to replace the use of megaprosthesis, but the development of high-performance scaffolds has the potential to treat large bone defects.

Nanofibers are gaining considerable attention as a scaffolding material for regenerative medicine [36]. A key characteristic of nanofiber scaffolds is their ability to change according to the adhesive and proliferative properties of cells by controlling their material and structure [37]. Various types of nanofibers are being researched as versatile scaffolding materials for bone regeneration and their use as void fillers in bone defects, which include carbon, natural polymers and synthetic polymers, as well as their composite material [38].

Electrospinning has been suggested as a method to produce nanofibers [39]. In the electrospinning system, the webs of nanosized fibers are obtained by applying a high voltage to the needle tip containing a polymer solution and evaluated as practical biomaterials for use in the clinical field. A variety of nanofibers have been synthesized via the electrospinning system and their safety and effectiveness as biomaterials has been evaluated (Table 1).

In this review, we introduce the effects and potential clinical applications of electrospun nanofibers in bone regenerative medicine, with a focus on carbon nanofibers.

## 2. Carbon Nanofibers

Carbon is abundant as an organic substance in the living body and demonstrates excellent biocompatibility, and the substance is already used as a biomaterial in clinical contexts, such as its use in artificial valves and coronary stents [40,41]. Although carbon fibers have been used as biological substitutes in the field of musculoskeletal medicine for repairing ligaments and tendons [42,43,44,45], there are no reports on their use as scaffolds. Research has been conducted on scaffolds for bones and cartilage, but such scaffolds have not reached clinical use [43,44].

**Figure 1:**
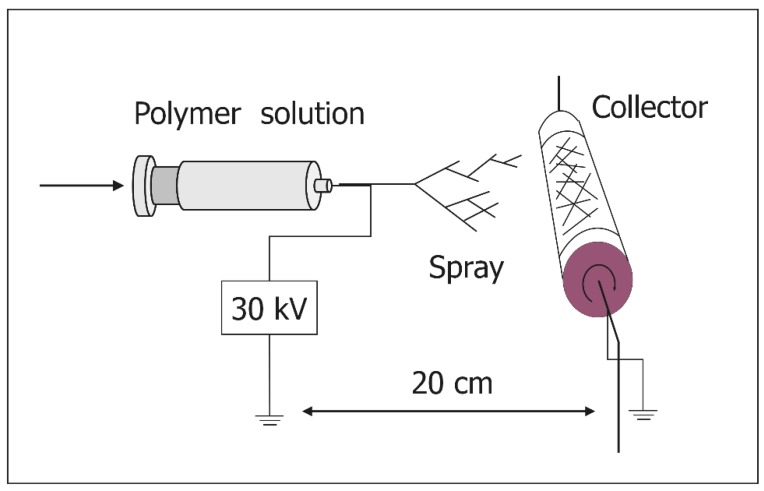
A schematic diagram of the electrospinning system for producing a thin carbon nanofiber web. The polyacrylonitrile solution was ejected from the needle tip under a high electric field and subsequently deposited in a form of web on collector.

**Figure 2:**
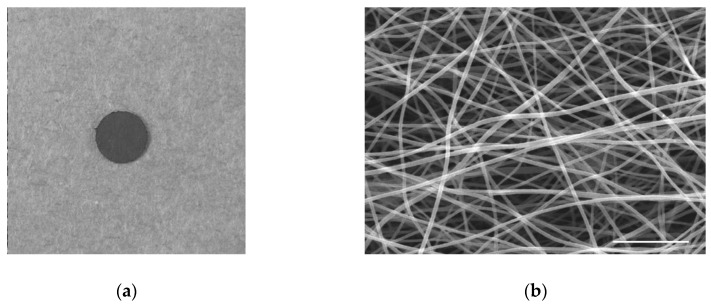
250 nm type thin carbon nanofiber web (TCFW). (**a**) Macroscopic view of implant for animal testing (diameter: 5 mm); (**b**) Scanning electron microscopy image. Nanofibers are presented as web structures. Scale bar, 5 μm.

We synthesized a flexible 3D thin carbon nanofiber web (TCFW) by electrospinning polyacrylonitrile and subsequent thermal treatment at 1000 °C in argon [46] (Figure 1). Two types of TCFWs with diameters of 250 and 1000 nm were evaluated as bone tissue scaffolds [47] (Figure 2).

According to X-ray photoelectron spectroscopy, the chemical composition of TWCFs with diameters of 250 and 1000 nm were 95.4% carbon, 3.8% nitrogen, 2.8% oxygen. Both samples showed high carbon purity with no noticeable impurity.

**Figure 3:**
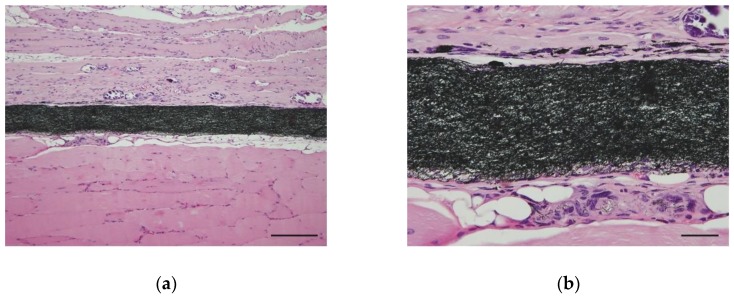
Optical microscope image of a 250 nm type TCFW that was implanted into the back muscle of a mouse for 4 weeks. (**a**) Fibroblasts and macrophages accumulated around the implants in the muscle tissue, forming foreign-body granulomas. No necrosis or strong inflammatory reactions were observed. Scale bar, 100 μm; (**b**) In the high-power field of view, nanofibers phagocytosed by macrophages were observed in the periphery of the implant. Scale bar, 20 μm.

In order to evaluate the biocompatibility of TCFW, we inserted a 5 mm diameter, disc-shaped implant into the back of ddY mouse (Japan SLC, Inc., Hamamatsu, Japan). Tissues were evaluated by hematoxylin- and eosin-staining at 4 weeks after transplantation. No apparent necrosis or strong inflammatory responses were observed around the TCFW implant. Macrophages and fibroblasts accumulated in the periphery of the implant, and we observed fragmented carbon nanofibers that were phagocytosed by macrophages (Figure 3).

**Figure 4:**
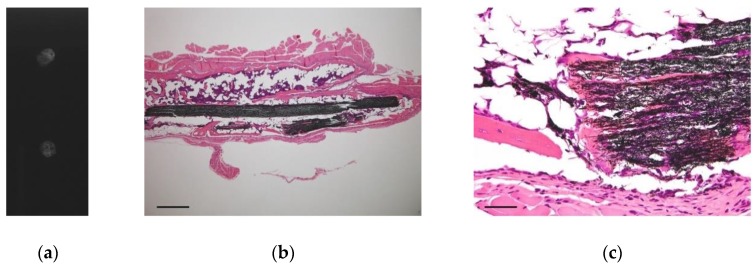
Ectopic bone formed from a rhBMP-2-loaded scaffold in a 250 nm type TCFW implant under the spinal muscle of the mouse. (**a**) Soft X-ray image of ectopic bone. Calcification occurred in the implant, and the trabecular structure is observed; (**b**) Low-power field optical microscopic image of ectopic bone. Bone matrices and bone marrow tissues are formed around the 250 nm type TCFW. Scale bar, 200 μm; (**c**) High-power field optical microscopic image of ectopic bone. Bone matrices are formed between the nanofibers of the implant. Scale bar, 20 μm.

Next, we evaluated the efficacy of TCFW as a scaffold for bone regeneration. Recombinant human bone morphogenetic protein-2 (rhBMP-2) was used as a growth factor for the cells. Because TCFW is hydrophobic, we used rhBMP-2 containing a biocompatible surfactant (0.01% Tween 80). A freeze-dried implant containing rhBMP-2 of the same shape that was inserted into the back muscle in the biocompatibility test was implanted into the lumbodorsal fascia of ddY mouse. At 3 weeks after implantation, the ectopic bone formation in the back muscle was harvested, and the pathological specimen was microscopically evaluated under soft X-ray imaging and hematoxylin- and eosin-staining. In the soft X-ray image, trabecular structures could be confirmed in the ectopic bone formed on the back of the mouse (Figure 4a). In histopathological specimens, the formation of bone matrices and bone marrow structures were observed around the TCFW implant (Figure 4b). We were able to observe that carbon nanofibers were integrated into the bone matrix and bone marrow cells in the high-power field of view (Figure 4c). Although carbon fiber is a non-biodegradable material, we believe that nano-sized carbon fiber can be incorporated into the bone matrix as a filler, and can potentially improve the mechanical strength of bone.

## 3. Natural Polymer Nanofibers

Collagen is abundantly found in the body, and over 90% of proteins in the bone matrix are comprised of collagen. In the development and regeneration of bone, the bone matrix is formed by mineral deposition onto a type I collagen that is produced by osteoblasts [48]. Collagen plays a key role in the development and regeneration of bone and has been studied as a scaffolding material for regenerative medicine. In animal models, collagen has been used as a scaffold for the regeneration of unloaded bone, such as the maxilla and mandible [49].

Research is also being conducted into the use of the electrospun collagen nanofiber as a scaffold for bone regeneration. Venugopal et al. developed a composite material composed of Type I collagen and HA produced via electrospinning [50]. The mean diameter of the collagen/HA nanofiber is 293 ± 1.45 nm, and the material demonstrates a good proliferation of human fetal osteoblast cells on the scaffold. The Alizarin Red S staining protocol for calcium [51] showed better calcification compared to that of collagen nanofiber scaffolds without HA. Lee et al. developed a composite material consisting of collagen fiber and polycaprolactone (PCL) with an approximate diameter of 350 nm [52]. Collagen nanofibers demonstrate excellent cellular adhesion and proliferative properties, and the mechanical strength of the scaffold was augmented by solidifying the collagen nanofibers with PCL. The authors cultured osteoblast-like cells (MG63) on this scaffold and measured an MTT (3-(4,5-dimethylthiazol-2-yl)-2,5-diphenyl tetrazolium bromide) assay to evaluate cell proliferation [53], which showed better cell proliferation compared to the PCL scaffold. Yeo et al. created a porous three-dimensional composite with β-tricalcium phosphate (βTCP) and PCL, which are components of the bone matrix, and filled the pores of the scaffold with collagen nanofibers with a diameter of 160 ± 80 nm using the electrospinning method [54]. In this scaffold, the MTT assay with MG63 cells showed a better cell proliferation compared to the βTCP/PCL scaffold without collagen nanofibers.

Although the use of collagen scaffolds is a gold standard in regenerative medicine, there have been attempts to use other natural polymers as scaffolds. Cellulose is a component of plant fiber and is used as a raw material for paper. Cellulose has excellent biocompatibility [55]. A composite of chitosan/cellulose was used as a gauze as a barrier for preventing postoperative adhesion after abdominal surgery [56], and methyl cellulose [57] was studied for its use as a scaffold in cartilage regeneration. Trivedi et al. cultured human osteoblasts on chitosan/cellulose hydrogel beads and demonstrated their potential as a scaffold for bone regeneration [58]. Chakraborty et al. produced a web-like cellulose scaffold with a diameter of from 300 to 600 nm by using the electrospinning method [59]. They cultured MC3T3-E1 osteoblast cells on the scaffold and reported good results using an MTT assay and scanning electron microscopy (SEM) for evaluation. In addition, Gasparic et al. produced a nanoparticle composite of cellulose and HA using the electrospinning method [60]. The electrospun nanofibers were several hundred nanometers in diameter and human-bone-derived osteoblasts were cultured on the scaffold, which demonstrated cell proliferation by MTT assay.

As a composite containing cellulose, the aforementioned chitosan is also used as a biomaterial. Chitosan is a natural material that can be obtained by the deacetylation of chitin, which is extracted from the exoskeleton of crustaceans such as crab and shrimp [61]. Chitosan is studied in the field of nerve regeneration [62] and skin regeneration [63]. In bone regeneration, Sharifi et al. produced electrospun nanofibers from a PCL and chitosan composite and produced a scaffold comprising of fibers with a diameter of from 350 to 500 nm [64]. The MTT assay was performed with MG63 cells on the scaffold to evaluate the cell viability. Liu et al. produced a scaffold from electrospun chitosan with HA nanoparticles which have diameters ranging between 200 and 300 nm [65]. The authors cultured bone-marrow-derived mesenchymal stem cells (BMSC) and performed evaluations using bone-specific alkaline phosphatase (ALP) staining. The HA/chitosan nanofiber scaffold showed better stainability compared to the HA/chitosan composite membrane. In addition, the repair of a rat critical-size calvarial defect was observed after filling the bone defect with the HA/chitosan nanofiber scaffold.

A summary of the literature discussed in this section are shown in Table 2.

## 4. Synthetic Polymer Nanofibers

In regenerative medicine research, the now-infamous experiment performed by Lenger, Vacanti, and their colleagues on what appeared to be a human ear grown on the back of a nude mouse made “regenerative medicine” a familiar term to the general public [66,67]. In this experiment, chondrocytes isolated from bovine articular cartilage were seeded onto a synthetic polymer template made of polyglycolic acid–polylactic acid in the shape of a human ear. The chondrocytes were subcutaneously implanted and grown in the dorsal region of an immunodeficient nude mouse that did not elicit a rejection response. In the field of bone tissue regeneration, Saito et al. developed a biodegradable polymer, poly lactic acid-p-dioxanone-polyethylene glycol block copolymer (PLA-DX-PEG), which was combined with rhBMP-2 as a growth factor [68]. The polymer was used to repair a pelvic bone defect in a rat model and demonstrated that synthetic polymers can be used as a scaffold for bone regeneration therapy. More recently, the potential clinical uses of poly (lactic-co-glycolic acid) (PLGA) have been evaluated as scaffolds for bone regeneration on defects of non-loading bones such as the maxilla and mandible, and excellent results have been reported in animal experiments [69].

In recent years, various synthetic polymers have been developed into nanofibers by the electrospinning method and evaluated as a scaffold material for regenerative medicine. As a composite of collagen nanofiber, there is a study on developing PCL as nanofibers by the electrospinning method and evaluating its use as a scaffold. Wang et al. fabricated electrospun PCL that was loaded with nanosilicate, and developed a scaffold consisting of nanofibers with a diameter of several hundred nanometers [70]. In the osteoblast cell line MC3T3-E1 cell culture, the cell viability and ALP activity increased with a dose dependency on PCL with nanosilicates. In an in vivo experiment that subcutaneously transplanted MC3T3-E1 cells that were cultured onto a nanosilicate/PCL scaffold and implanted into the back of rats, the nanosilicate/PCL scaffold presented a stronger expression of osteocalcin (OCN) [71], which is a bone formation marker, compared to the PCL scaffold at 4 weeks postoperatively. Yang et al. developed and reported an eletrospun scaffold that incorporated nanosilicates into PLGA [72]. A scaffold consisting of nanofibers with diameters of approximately 500 to 800 nm were obtained and osteoblast-like cells (SaOS-2 cells) were cultured on the scaffold. Alizarin Red S staining and ALP activity were evaluated, and showed that the nanosilicate/PLGA scaffold promotes better bone differentiation compared to the PLGA scaffold. Enayati et al. developed a PVA/HA scaffold that incorporated poly (vinyl alcohol) (PVA) with HA nanoparticles using the electrospinning method [73]. The diameter of this nanofiber was approximately 150 nm and, in the MTT assay of MG63 cells, there was no significant difference in cell viability with the PVA scaffold without HA. However, the PVA/HA scaffold exhibited better results in terms of Alizarin Red S staining and ALP activity, and the scaffold was found to facilitate the differentiation of osteoblasts. Zhang et al. fabricated a layer of nanofibers with poly-L-lactic acid (PLA) by electrospinning, attached an additional layer of freeze-dried collagen, and created a bi-layer collagen/PLA scaffold [74]. A stronger OCN gene expression was observed in the BMSC cultured on this scaffold compared to the BMSC cultured on the collagen scaffold. Moreover, the group injected with the collagen/PLA scaffold on the osteochondral defect created on the distal articular surface of the femur of the rabbit exhibited a better regeneration of cartilage bone compared to the collagen scaffold group, according to the Visual Histological Assessment Scale of the International Cartilage Repair Society [75].

A summary of the literature discussed in this section is also shown in Table 2.

## 5. Cells for Bone Regeneration and Signaling Molecules

Aside from scaffolds, cells and signaling molecules are also of importance in regenerative medicine. The same is true for bone regeneration and, by using them, the efficiency of bone regeneration can be improved. At present, synthetic bone substitutes made of calcium phosphate (βTCP, HA) are widely used scaffolds for the clinical treatment of bone defects. Although these materials exhibit good osteoconductivity for smaller bone defects, there is a limitation in terms of the size of the bone defect that can be treated [12]. Therefore, to increase the efficiency of bone regeneration therapy, treatments that combine the use of scaffolds with various cells and signaling molecules are being studied.

In recent years, there has been considerable interest in iPS cells for their potential use in scaffolds [33,34]. iPS cells are multipotent cells that can differentiate into various cells in the body and are made by introducing several types of genes known as Yamanaka factors into somatic cells, such as skin cells. These cells have generated considerable excitement for their potential use in regenerative medicine and the treatment of intractable diseases. Studies have also been conducted on the clinical application of iPS cells for seeding on nanofiber scaffolds in bone regeneration [76,77].

Specifically, in terms of bone regeneration, cells seeded on scaffolds do not need to be as multipotent as iPS cells. Before the advent of iPS cells, research on bone-marrow-derived stem cells (BMSC) was conducted [78,79]. In clinical practice, BMSCs can be obtained less invasively from the iliac bone marrow with relative ease, and can be differentiated into osteoblast precursor cells by culturing in an osteogenic medium containing β-glycerophosphate or dexamethasone [80]. Research has been conducted to cultivate BMSC on scaffolds to differentiate into osteoblast progenitor cells and promote bone formation. Liu et al. reported on a chitosan/HA composite [66], while Xu et al. reported on a PCL/PLLA composite [81] that combines nanofibers and BMSC for bone regeneration.

BMP-2 is the most well-known signaling molecule. BMP-2 exerts a potent bone anabolic effect and has been clinically applied for treating fractures and bone loss [82,83]. Scaffolds for osteogenesis also serve as carriers in the drug delivery system (DDS) of BMP-2, which is a powerful signaling molecule. We were able to obtain good bone formation using BMP-2 as a signaling molecule for carbon nanofiber scaffold. In addition, there are also studies on BMP-2 as a signaling molecule for biodegradable scaffolds [84,85].

For other signaling molecules, vascular endothelial growth factors and BMPs such as BMP-6 and BMP-7 are attracting attention in regenerative medicine as angiogenic factors for clinical application [86,87]. Aside from cultured cells, a method of combining platelet-rich plasma (PRP) with scaffolds is also being studied. PRP contains an abundance of growth factors such as VEGF, insulin-like growth factor (IGF), platelet-derived growth factor (PDGF), and transforming growth factor beta (TGF-β). These growth factors do not act as cells that differentiate into tissues, but rather serve as a DDS for signaling molecules [88,89]. Using a silk fibroin/PCL composite implant with PRP that was fabricated using electrospinning, Cheng et al. repaired critical-sized calvarial bone defects in rats [90].

The characteristics required in scaffolds for bone tissue include the adhesiveness of induced cells for localized seeding and sustained release of signaling molecules. The appropriate type, thickness, length, pore size, and three-dimensional structure of the fiber for bone regeneration are being studied [91,92,93]. However, further studies must be conducted to determine the optimal structure for bone tissue regeneration. We may be able to develop scaffolds that can repair larger bone defects without the use of signaling molecules or cells, provided that the optimal conditions for bone tissue regeneration are clarified.

## 6. Biological Safety of Nanofibers

Biocompatibility and toxicity are important issues for biomaterials that are implanted and used in living bodies. For nanomaterials, in addition to the properties of the material itself, their size and form must also be considered for safety. Titanium dioxide is widely used as a pigment, paint, and food additive; however, the nanoparticles of titanium dioxide may be carcinogenic, and studies have continued to evaluate their safety [94]. In a study comparing the inflammatory responses of titanium dioxide with nano-sized and submicron-sized particles when exposed during respiratory inhalation, animals inhaling nano-sized particles had a stronger inflammatory response [95,96]. Thus, with nano-sized materials, biological response may vary according to the size and shape of the same substance.

We evaluated two types of TCFW with a diameter of 1000 nm and 250 nm, and evaluated the biocompatibility and performance of the material as scaffolds for bone tissue [47]. The biocompatibility with the mouse muscle was good in both the 250 and 1000 nm type TCFW, and bone formation was also observed in the ectopic osteogenesis experiment using rhBMP-2. Usui et al. reported good bone formation using a multi-walled carbon nanotube (MWCNT) with a diameter of approximately 80 nm, although MWCNT is not a nanofiber fabricated by the electrospinning method [97]. Vittorio et al. showed that MWCNT with a higher purity produces a better cellular affinity than MWCNT with a lower purity [98]. Carbon materials are generally thought to have excellent compatibility with bone tissue. However, attention should be paid to impurities that are introduced in the manufacturing process.

In terms of biodegradable nanofibers, the biocompatibility of the material is more important than its structure. In this review, the nanofibers that were introduced as examples exhibited excellent biocompatibility; however, further attention must be paid to the biocompatibility and toxicity in terms of their impurities and composite materials [99]. In addition, immune responses such as disease transfer and xenogenicity should be carefully considered for natural polymers such as collagen, cellulose, and chitosan before their use in clinical applications [100,101,102].

## 7. Conclusions

Nanofibers that are fabricated by electrospinning have been evaluated and studied as scaffolds for regenerative medicine. Various nanofibers are also being studied as scaffolds for bone regeneration. Research continues to be conducted on carbon nanofibers, natural polymers, synthetic polymers, and their composite nanofibers. Although the scaffolds of these nanofibers are not in clinical use, we believe that excellent scaffolds for bone tissue regeneration may be developed by taking advantage of their structure and function. In order to discover the clinical applications of electrospun nanofiber scaffolds for bone regeneration medicine, further studies should be conducted on their structure, biological safety, and combined use with signaling molecules.

## Figures and Tables

**Table 1:** Elecrospun nanofiber scaffolds.

Non-Biodegradable		Carbon
Biodegradable	Natural polymer	Collagen, cellulose, chitosan, etc.
	Synthetic polymer	PCL, PLA, PLGA, PVA, etc.

PCL: polycaprolactone, PLA: poly-L-lactic acid, PLGA: poly (lactic-*co*-glycolic acid), PVA: poly (vinyl alcohol).

**Table 2:** The literature on electrospun nanofiber-based scaffolds.

Author, Year	Material	Composite	Diameter (nm)	Cells/Animals	Signaling Molecules	Evaluations
Present study	carbon	-	250	ddY mouse	rhBMP-2	ectopic bone
Venugopal et al., 2008 [50]	collagen	HA	293	human fetal osteoblast cell	-	Alizarin Red S stain
Lee et al., 2008 [52]	collagen	PCL	350	MG63 cell	-	MTT assay
Yeo et al., 2011 [54]	collagen	βTCP/PCL	160 ± 80	MG63 cell	-	MTT assay
Chakraborty et al., 2019 [59]	cellulose	-	300–600	MC3T3-E1 cell	-	MTT assay SEM
Gasparic et al., 2017 [60]	cellulose	HA	several hundred	human bone derived osteoblast	-	MTT assay
Sharifi et al., 2018 [64]	chitosan	PCL	350–500	MG63 cell	-	MTT assay
Liu et al., 2013 [65]	chitosan	HA	200–300	BMSC SD rat	BMSC	ALP stain cranial bone defect
Wang et al., 2018 [70]	PCL	nanosilicate	several hundred	MC3T3-E1 cell SD rat	MC3T3-E1 cell	ALP activity OCN expression
Yang et al., 2018 [72]	PLGA	nanosilicate	500–800	osteoblast-like cells (SaOS-2 cells)	-	Alizarin Red S stain
Enayati et al., 2018 [73]	PVA	HA	150	MG63 cell	-	Alizarin Red S stain, ALP activity
Zhang et al., 2013 [74]	PLA	Collagen	data not shown	BMSCrabbit	-	OCN gene expression femur osteochondral defect

rhBMP-2: human bone morphogenetic protein-2, HA: hydroxyapatite, MTT: 3-(4,5-dimethylthiazol-2-yl)-2,5-diphenyl tetrazolium bromide, βTCP: β-tricalcium phosphate, SEM: scanning electron microscopy, BMSC: bone marrow-derived mesenchymal stem cell, ALP: alkaline phosphatase, SD: Sprague-Dawley, OCN: osteocalcin.
